# Intracystic papillary neoplasm diagnosis following an extended cholecystectomy: a case report … and literature review

**DOI:** 10.1093/omcr/omad051

**Published:** 2023-06-26

**Authors:** Nao Kitasaki, Masashi Inoue, Tomoyuki Abe, Akihiro Kohata, Masatoshi Kochi, Ryuichi Hotta, Tsuyoshi Kobayashi, Hideki Ohdan, Kazuhiro Toyota, Tadateru Takahashi

**Affiliations:** Department of Gastroenterological and Surgery, National Hospital Organization Higashihiroshima Medical Center, Hiroshima, Japan; Department of Gastroenterological and Surgery, National Hospital Organization Higashihiroshima Medical Center, Hiroshima, Japan; Department of Gastroenterological and Surgery, National Hospital Organization Higashihiroshima Medical Center, Hiroshima, Japan; Department of Gastroenterological and Surgery, National Hospital Organization Higashihiroshima Medical Center, Hiroshima, Japan; Department of Gastroenterological and Surgery, National Hospital Organization Higashihiroshima Medical Center, Hiroshima, Japan; Department of Gastroenterological and Surgery, National Hospital Organization Higashihiroshima Medical Center, Hiroshima, Japan; Department of Gastroenterological and Transplant Surgery, Graduate School of Biomedical and Health Sciences, Hiroshima University, Hiroshima, Japan; Department of Gastroenterological and Transplant Surgery, Graduate School of Biomedical and Health Sciences, Hiroshima University, Hiroshima, Japan; Department of Gastroenterological and Surgery, National Hospital Organization Higashihiroshima Medical Center, Hiroshima, Japan; Department of Gastroenterological and Surgery, National Hospital Organization Higashihiroshima Medical Center, Hiroshima, Japan

## Abstract

We report a case of intracystic papillary neoplasms (ICPN) that was difficult to differentiate from adenocarcinoma of the gallbladder. A 64-year-old man visited our hospital for an examination of gallbladder tumors. At the preoperative examination, the tumor was revealed a papillary type of tumor in the body of the gallbladder without the findings that without the findings that suggested the tumor invasion into the deep subserosal layer. The patient underwent an extended cholecystectomy. Papillary lesions were observed mainly in the body of the gallbladder, with flattened elevated lesions at the gallbladder fundus. Within each of these tumors, cells corresponding to intraepithelial adenocarcinoma were irregularly interspersed, leading to a diagnosis of ICPN. The patient is currently undergoing follow-up with no recurrence postoperatively. The prognosis of ICPN is generally good; however, preoperative diagnosis remains challenging. Therefore, a treatment plan for gallbladder cancer should be applied.

## INTRODUCTION

Intracystic papillary neoplasms (ICPN) was first defined in the 2010 World Health Organization classification [1] as a tumor of the gallbladder composed of dysplastic cells and is recognized as the cholecystic counterpart to intraductal papillary mucinous neoplasms of the pancreas (IPMN) and intraductal papillary neoplasms of the bile duct [2]. It may include a variety of previously reported terminology, such as papillary adenoma, papillary *in situ* carcinoma and papillary adenocarcinoma of the gallbladder. ICPN is more common in women over 60 years of age and has been reported in <0.5% of the gallbladders removed because of cholelithiasis or chronic cholecystitis, but the imaging characteristics remain unclear [2, 3]. Herein, we report a case of ICPN with literature review.

## CASE REPORT

**Figure 1:**
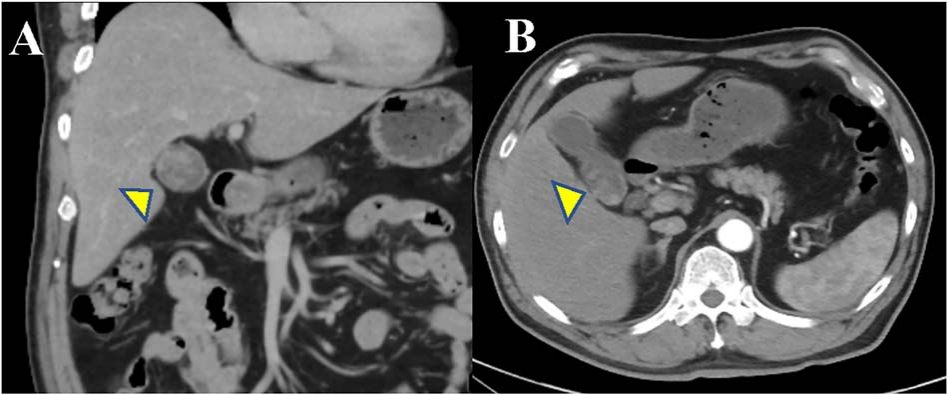
Abdominal CT findings. (**A**, **B**) The arterial phase shows a slightly enhanced low-density tumor, maximum of 27 mm in size, extending from the body to the bottom of the gallbladder.

**Figure 2:**
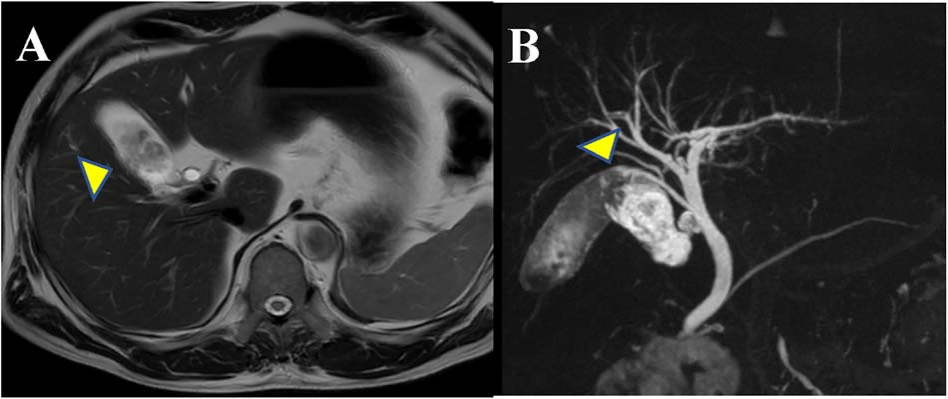
Findings of magnetic resonance imaging (MRI): (**A**) T2-weighted MRI (coronal image) shows the papillary tumor filling the body of the gallbladder. (**B**) T2-weighted MRCP image shows the accessory hepatic duct and it joins the common bile duct dorsally in the middle of the common bile duct.

**Figure 3:**
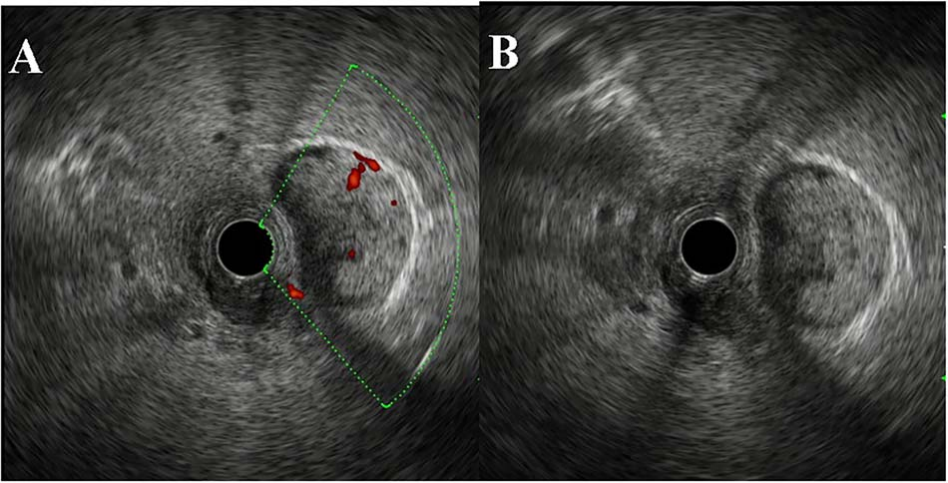
EUS findings. (**A**, **B**) EUS demonstrated polypoid lesions extending from the gallbladder without invasion of the gallbladder wall or the bile duct. Increased blood flow was seen in the neck of the tumor.

**Figure 4:**
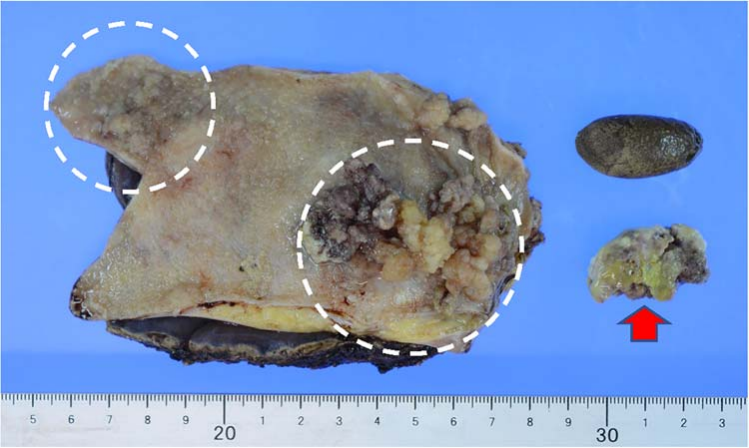
Imaging: the gallbladder mucosa was multifocal with a papillary tumor measuring 65 × 36 mm in the body of the gallbladder and a flat-expanding type tumor at the gallbladder fundus. Tumors that were partially dissected during specimen preparation showed similar findings. (➡) Gallstones were also contained within the gallbladder.

**Figure 5:**
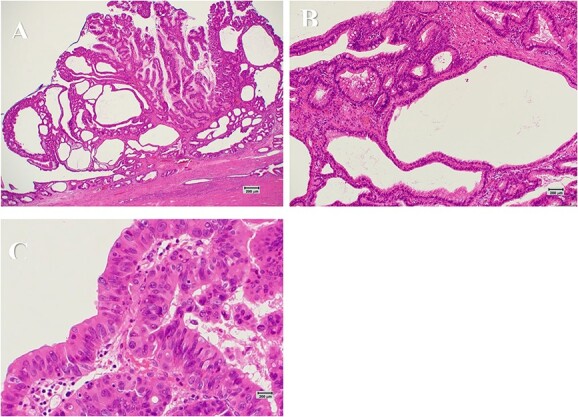
Histopathological findings (scar: 1 cm = 200 μm). (**A**) The tumor shows a papillary growth of the epithelium with delicate fibrovascular stalks (HE stain ×40). (**B**) The papillary lesion is composed mostly of columnar epithelial cells having intracytoplasmic mucin and basally located small nuclei (HE stain ×200). (**C**) A part of epithelial cells is severely atypical with loss of polarity, suggestive of adenocarcinoma (HE stain ×400).

**Figure 6:**
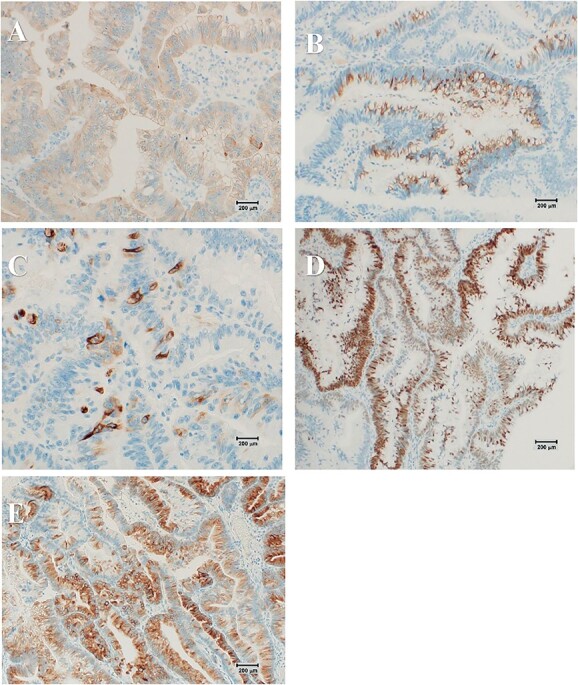
(Scar: 1 cm = 200 μm) Immunohistochemical analysis of the tumor shows positive staining for MUC2 (**B**), MUC5AC (**C**) and CDX2 (**D**) but negative staining for MUC1 (**A**) and MUC6 (**E**), suggesting the intestinal type.

A 64-year-old man visited our hospital for an examination of a gallbladder tumor discovered incidentally during an evaluation of liver dysfunction. His medical history included diabetes mellitus and fatty liver. Laboratory data demonstrated that elevated hepatic enzymes and the normal limits of carcinoembryonic antigen and carbohydrate antigen 19–9 levels. Computed tomography revealed a tumor measuring up to 27 mm in diameter, spanning from the neck to the body of the gallbladder. A contrast effect was also observed in the early phase without significant lymph node enlargement (Fig. 1A and B). Magnetic resonance cholangiopancreatography revealed a papillary tumor filling the body of the gallbladder (Fig. 2A and B). Endoscopic ultrasound detected increased blood flow to the tumor without the findings of tumor invasion into the deep subserosal layer (Fig. 3A and B). Bile cytology showed no malignant findings. Therefore, gallbladder cancer with an extensive basal spread of blood flow and ICPN with adenocarcinoma were suspected. The patient underwent extended cholecystectomy (ExCC). Macroscopically, the papillary lesions were observed primarily in the body of the gallbladder, measuring 65 × 36 × 12 mm, with flattened elevated lesions scattered at the gallbladder fundus (Fig. 4). The tumor cells were mainly atypical adenoma-like cells and were heterogeneously distributed corresponding to intraepithelial adenocarcinoma. However, no evidence of stromal invasion was observed. Similar findings were observed for a flattened elevated lesion at the base of the gallbladder, both of which led to the diagnosis of ICPN (Fig. 5). Immunohistochemical analysis of the tumor suggested the intestinal type (Fig. 6). The above-described immunohistochemical patterns were seen only within the ICPN area. The patient is currently undergoing follow-up without any postoperative recurrence observed in the last 6 months. We will continue to perform blood tests and imaging tests every 6 months up to 5 years postoperatively.

## DISCUSSION

ICPN is a rare clinicopathologic entity and a relatively newly defined disease. Although 50% of the patients with ICPN have an invasive malignant component, the prognosis for ICPN is good: 3-year survival averages 90% for lesions with no foci of invasion versus 60% for those with foci of invasion [2].

IPMN has a well-defined disease concept and several studies have demonstrated its associated risk with malignant transformation [4, 5]. Furthermore, the indicators for surgical resection are well-established in the literature and its natural history remains well-known [6, 7]. However, the imaging characteristics of ICPNs are not well established. According to Mizobuchi *et al*. [8], the typical imaging presentation of ICPN is a large papillary polypoid lesion with contrast in the gallbladder, without deformity or extrinsic progression. Furthermore, ICPN tends to be enhanced in the early phase and remains enhanced in the delayed phase. There have been cases of ICPN that have remained observable over a 2-year period. On the other hand, gallbladder tumors with contrast enhancement and polypoid lesions of large size, as in this case, require aggressive resection.

There are four histologic subtypes of ICPN [2]. The frequency of pancreatobiliary and oncocytic types is low; they are highly malignant and have a poor prognosis, especially the pancreatobiliary type that is frequently associated with invasion [2]. Among invasive cases, MUC1-positive cases have a poor prognosis and are known to have a high frequency of lymph node metastases. Therefore, the diagnosis of histological subtypes may be useful for estimating treatment prognosis. In this case, immunohistochemical analysis of the tumor revealed that MUC2 and MUC5AC were positive and MUC1 and MUC6 were negative, resulting in the intestinal subtype classification. Our case showed MUC1-negative result, and no findings with invasion of the subserosal layer were noted; thus, the patient was followed up without the requirement for chemotherapy.

We searched PubMed and the Journal of Health Care and Society from January 2014 to March 2022 and identified a total of 26 case reports (Table 1). Laparoscopic cholecystectomy (LC) was selected for those with a preoperative diagnosis of ICPN, whereas the choice of surgical technique in other cases was based on the presence or absence of invasion and localization. ExCC was performed in 15 cases, and pancreaticoduodenectomy in four cases. There were 15 cases in which lymph node dissection was omitted, but all of them had no recurrence. Although invasive carcinoma requires extensive surgery including lymph node dissection, only two cases with invasive components were found during the review, both of which underwent lymph node dissection, and only one of the two cases recurred. Kang *et al*. [9] reported that ICPN with invasive carcinoma were more often resected at an early stage compared with conventional gallbladder cancer and had a better prognosis; however, the prognosis was similar when T-stage matching was performed. This trend was also observed in the present review, and it seems reasonable to adopt the same treatment strategy for ICPN with invasion as for conventional gallbladder cancer. At present, it is impossible to distinguish invasive from noninvasive carcinoma by diagnostic imaging, and many centers perform an ExCC with a frozen section, as in this case. If a more accurate preoperative diagnosis is possible, minimally invasive treatment with LC combining diagnosis and treatment, followed by a two-stage resection, if necessary, will be possible.

**Table 1:** Clinical characteristics of patients with ICPN

	Report year	Author	Year	Sex	CEA (ng/mL)	CA19–9 (U/mL)	Imaging	Preoperative diagnosis	Preoperative invasion	Localization	Operative method	Pathological invasion	Subtype	Outcome
1	2014	Hashimoto	58	F	N/A	185	Irregular wall thickening	Gbc	Negative	the cystic duct	PD + ExCC	subserosa layer	biliary type	1 year 4 month, liver metastasis
2	2014	Meguro	54	F	N/A	97	Papillary tumor and nodular tumor	Mucin-producing neoplasm with pancreaticobiliary malijunction	Negative	N/A	ExCC + Bile duct resection	muscular layer	biliary type + LCNEC	1 year no metastasis
3	2016	Sakamoto	63	M	6.2	<37	Papillary tumor	IPNB	Negative	the cystic duct	ExtCC + Bile duct resection	fibromuscular layer	biliary type	2 year 10 month no metastasis
4	2016	Sato	64	M	2	2.9	Cystic tumor	ICPN	Negative	the gallbladder fundus	LC	fibromuscular layer	intestinal type	8 months no metastasis
5	2017	Kaino	74	M	2.1	7.4	Papillary tumor	GbC	Negative	the gallbladder fundus	ExCC	fibromuscular layer	biliary type	3 months no metastasis
6	2018	Shiozawa	70	F	16.2	369	Papillary tumor	gallbladder cancer of papillary infiltrating type	Liver	the body of the gallbladder	Right liver resection + Bile duct resection	N/A	intestinal type	5 years 4 months no metastasis
7	2018	Muranushi	70	M	4.4	10.4	Irregular wall thickening	GbC	Negative	the gallbladder fundus	ExCC	N/A	biliary type	3 years 6 months no metastasis
8	2018	Futai	78	F	1.1	2.33	Papillary tumor	GbC	Negative	the gallbladder fundus	ExCC	mucosa layer	oncocytic type > gastric type	N/A
9	2018	Mizobuchi	74	F	5.4	82.1	Papillary tumor	GbC	Negative	the gallbladder fundus	ExCC	mucosa layer	gastric type	N/A
10	2018	Mizobuchi	61	F	1	<2.0	Papillary tumor	GbC	Negative	the gallbladder fundus	ExCC	N/A	intestinal type	N/A
11	2018	Mizobuchi	83	M	3.5	41.4	Papillary tumor	GbC	Negative	the gallbladder fundus	ExCC	N/A	N/A	N/A
12	2019	Fujii	59	M	<5	<37	Papillary tumor	primary cystic duct cancer	Extensive bile ducts	bile duct~common bile duct	PD	mucosa layer	gastric type>biliary type	2 months no metastasis
13	2019	Yokode	58	F	<5	<37	Papillary tumor	primary cystic duct cancer	Negative	bile duct~common bile duct	PD	subserosa layer	gastric type	6 months no metastasis
14	2020	Oh	79	F	N/A	N/A	Papillary tumor	ICPN	Negative	the body of the gallbladder	LC	N//A	N/A	3 years no metastasis
15	2020	Iwasaki	52	M	N/A	30	Papillary tumor	Gbc	Negative	the gallbladder fundus	ExCC + Bile duct resection	mucosa layer	gastric type	5 months no metastasis
16	2020	Sciarra	66	F	N/A	N/A	Papillary tumor	Gbc	Negative	N/A	ExCC	proper mucosal layer	N/A	N/A
17	2021	Oba	78	F	15.9	741	Papillary tumor	mucin-producing gallbladder tumor	Negative	the body of the gallbladder	LC	mucosa layer	intestinal type	2 years 6 months metastasis
18	2021	Logrado	71	F	<5	<37	Papillary tumor	ADM	Negative	the gallbladder fundus	LC	mucosa layer	intestinal type	1 years 8 months metastasis
19	2021	Iseki	83	M	3.3	94.3	Papillary tumor	tumor of the cystic duct and adenocarcinoma of the distal bile duct	Negative	the cystic duct and the distal bile duct	PD	fibromuscular layer	biliary type	6 months no metastasis
20	2021	Nakamura	71	M	3	1249.2	Papillary tumor	GbC	Negative	the body of the gallbladder	ExCC	N/A	biliary type or oncocytic type	N/A
21	2021	Aida	65	F	2.1	24	Papillary tumor and wall thickening	GbC	Liver	the gallbladder fundus	ExCC	mucosa layer	gastric type	3 months no metastasis
22	2021	Koike	44	M	N/A	N/A	Papillary tumor	a cholesterol polyp or pyliric-type adenoma	Negative	the body of the gallbladder	LC	mucosa layer	gastric type	N/A
23	2021	Shimada	69	M	2.1	8.5	Papillary tumor	GbC	Negative	the gallbladder fundus	ExCC	mucosa layer	N/A	1 year no metastasis
24	2022	Oishi	60	F	N/A	N/A	Papillary tumor	ICPN	Negative	the cystic duct	ExCC + Bile duct resection	mucosa layer	gastric type	2 years no metastasis
25	2022	Watanabe	79	F	N/A	N/A	Nodular tumor	Remnant GbC	Negative	the remnant gallbladder	Bile dust resection (after CC)	N/A	N/A	8 months no metastasis
26	2022	Our case	64	F	2.1	7.9	Papillary tumor	GbC	Negative	the body of the gallbladder	ExCC	mucosa layer	intestinal type	6 months no metastasis

N/A, not applicable; ADM, adenomyomatosis of gallbladder; GbC, gallbladder cancer; IPNB, intraductal papillary neoplasm of the bile duct; PD, pancreaticoduodenectomy; LCNEC, large cell neuroendocrine carcinoma; MiNEN, mixed neuroendocrine-non-neuroendocrine neoplasm

In conclusion, ICPN has a good prognosis; however, preoperative imaging is still challenging. Therefore, a treatment plan addressing gallbladder cancer should be employed. Additionally, only few cases have been reported, careful follow-up is essential.

## FUNDING

No funding body was involved in the design of the study and collection, analysis and interpretation of data and in writing the manuscript.

## ETHICS APPROVAL

All procedures used in this research were approved by the Ethical Committee of our institution.

## CONSENT

Written informed consent was obtained from the patient for the publication of this case report and accompanying images.

## CONFLICT OF INTEREST STATEMENT

None declared.

## DATA AVAILABILITY

Data sharing is not applicable to this article as no data sets were generated or analyzed during the current study.
